# Dynamics of Circulating Tumor DNA (ctDNA) Predict Treatment Efficacy and Prognosis in Patients With Advanced Pancreatic Cancer: A Prospective Large‐Cohort Study

**DOI:** 10.1002/mco2.70829

**Published:** 2026-06-23

**Authors:** Tingting You, Hui Tang, Mingming Yuan, Dongfeng Song, Chenyu Wang, Jinrong Ying, Rongrong Chen, Chunmei Bai, Yuejuan Cheng, Yingyi Wang

**Affiliations:** ^1^ Department of Medical Oncology Peking Union Medical College Hospital Chinese Academy of Medical Sciences Beijing China; ^2^ Department of Internal Medicine Peking Union Medical College Hospital Chinese Academy of Medical Sciences Beijing China; ^3^ Geneplus‐Beijing Beijing China

**Keywords:** advanced pancreatic cancer, ctDNA dynamics, treatment efficacy

## Abstract

Current methods for assessing treatment response and prognosis in pancreatic ductal adenocarcinoma (PDAC) have intrinsic limitations, necessitating novel biomarkers. This prospective cohort study evaluated whether early dynamic changes in ctDNA predict treatment efficacy and outcomes in advanced PDAC. We enrolled 127 patients receiving first‐line therapy, with peripheral blood collected at baseline (B1), first response assessment (B2), and subsequent timepoints for high‐depth next‐generation sequencing. The ctDNA detection rates significantly decreased from 78.7% at B1 to 55.7% at B2. Elevated CA19‐9 levels, ctDNA, and maximum somatic allele frequency (MSAF) were associated with disease progression. Higher B1 ctDNA and MSAF correlated with shorter progression‐free survival (PFS) and overall survival (OS), consistent with an external validation cohort. The Cox regression confirmed that MSAF and CA19‐9 were significant prognostic factors. In CA19‐9‐negative PDAC patients, increased ctDNA levels were associated with shorter PFS. This study demonstrated that high ctDNA levels and MSAF values are indicators of poor prognosis. Conversely, a decrease or clearance of ctDNA and MSAF suggests better disease control. In addition, in CA19‐9‐negative patients, dynamic ctDNA monitoring was able to indicate disease control and predict patient prognosis.

## Graphical abstract

A total of 127 advanced pancreatic ductal adenocarcinoma (PDAC) patients receiving first‐line therapy were enrolled in this study. A series of peripheral blood collected before the initiation of treatment (B1), at the first response assessment (B2), and at subsequent timepoints to perform high‐depth next‐generation sequencing. We underscore the potential of on‐treatment ctDNA monitoring as a dynamic and robust biomarker for predicting treatment response and patient outcomes in PDAC, thereby offering a more precise and personalized approach to clinical management and therapeutic decision‐making.

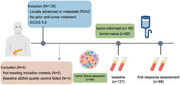

## 1 Introduction

Pancreatic cancer, known for its high mortality and extremely poor prognosis, accounted for 511,000 new cases and 467,000 deaths in 2022. It ranks as the sixth leading cause of cancer mortality and contributes to 4.8% of cancer‐related deaths worldwide [1]. Owing to its low early detection rate, high rates of recurrence and metastasis, and high resistance rate to medications, pancreatic cancer had the lowest 5‐year survival rate among all cancers in China from 2012 to 2015 [2]. Pancreatic ductal adenocarcinoma (PDAC) is the most prevalent pathological type of pancreatic cancer [3]. The majority of patients are diagnosed with advanced cancer, including locally advanced and metastatic PDAC (M‐PDAC), making them ineligible for curative surgery [4]. For unresectable PDAC, comprehensive treatment involving chemotherapy can alleviate symptoms, prolong survival, and improve quality of life; however, the majority of patients develop resistance to chemotherapy [4, 5, 6]. Therefore, accurate prediction of treatment efficacy and prognosis could avoid unnecessary drug toxicity and cost, and allow the choice of an alternative treatment regimen that might improve the clinical outcome.

The Response Evaluation Criteria in Solid Tumors (RECIST), which is primarily based on changes in tumor volume during treatment, has been routinely employed in clinical settings to assess the therapeutic response in advanced PDAC patients [7, 8]. However, the tumor microenvironment of PDAC is characterized by a dense stroma, which delays the detection of tumor shrinkage or growth through imaging techniques such as computed tomography (CT) and magnetic resonance imaging (MRI) [9, 10, 11]. Consequently, most patients are classified as having stable disease (SD) during early treatment assessments, and solely depending on changes in tumor volume may not reliably reflect the actual effectiveness of therapeutic interventions [12, 13]. Carbohydrate antigen 19‐9 (CA19‐9) is the most valuable serological biomarker for PDAC diagnosis, with a sensitivity of 78.2% and specificity of 82.8% when a threshold value of 37 U/mL is used [14]. Multiple studies have demonstrated an association between serum CA19‐9 levels and the recurrence or progression of PDAC following treatment, enabling the noninvasive assessment of treatment response [15, 16, 17, 18]. However, the utility of CA19‐9 has several limitations: approximately 20% of PDAC patients do not exhibit elevated CA19‐9 levels, and CA19‐9 levels can also be elevated in the case of inflammation or other malignancies [19, 20]. Therefore, the discovery of new biomarkers has become an urgent clinical necessity [21].

Liquid biopsies, including circulating tumor cells (CTCs), circulating tumor DNA (ctDNA), noncoding RNAs (ncRNAs), messenger RNAs (mRNAs), extracellular vesicles (EVs), and other components, have garnered significant attention from researchers due to their noninvasive nature and capacity for dynamic monitoring [21, 22, 23, 24]. ctDNA is an emerging noninvasive biomarker for prognostication and prediction of therapeutic response, and ctDNA‐based mutation detection has demonstrated its ability to reflect tumor burden and predict the risk of disease progression across multiple cancer types, including breast, lung, and gastric cancers [25, 26, 27, 28, 29, 30]. Several studies have investigated the potential of ctDNA in predicting chemotherapy efficacy and prognosis in advanced PDAC patients [31, 32, 33, 34, 35, 36, 37, 38]. However, these studies are largely limited by the use of small cohorts or the detection of only *KRAS* mutations. The employment of multi‐gene panel deep sequencing holds promise for overcoming tumor heterogeneity, improving the sensitivity of ctDNA detection, and guiding targeted therapies, thereby potentially informing therapeutic strategies and enhancing patient outcomes [21].

This prospective, large‐cohort study aims to ascertain whether early dynamic changes in ctDNA, as detected through high‐depth next‐generation sequencing (NGS), can predict treatment response and patient outcomes in patients with advanced PDAC. Furthermore, this study evaluated the clinical utility of dynamic ctDNA monitoring in patients with advanced pancreatic cancer, assessing its ability to predict patient prognosis and response to first‐line chemotherapy. To explore the real‐world clinical applications of ctDNA, this study further compared the detection rates of two different testing methods within this cohort and evaluated the clinical value of ctDNA in patients with negative CA19‐9 levels.

## 2 Results

### 2.1 Association Between B1 ctDNA and Clinical Characteristics

**FIGURE 1:**
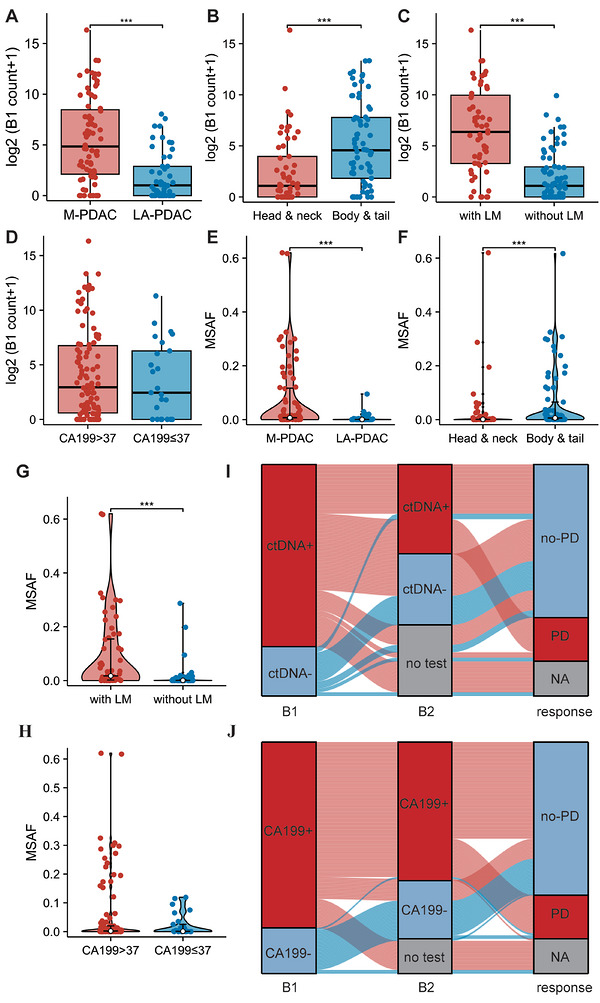
Relationships between clinical characteristics and baseline ctDNA. (A–D) Differences in baseline ctDNA levels in the M‐PDAC and LA‐PDAC cohorts (A), primary tumors located in the pancreatic head/neck or body/tail (B), with or without liver metastases (LM) (C), and baseline CA19‐9 status (D). (E–H) Differences in baseline ctDNA MSAF in the M‐PDAC and LA‐PDAC cohorts (E), primary tumor location (F), with or without LM (G), and baseline CA19‐9 status (H). The error bars represent the median with the interquartile range (IQR). (MSAF, maximum somatic allele frequency). (I and J) Sankey plot depicting changes in the proportion of patients stratified by ctDNA status (I), CA19‐9 status (J) and chemotherapy response.(*, *p* value < 0.05; **, *p* value < 0.01; ***, *p* value < 0.001).

Between January 2023 and January 2024, 136 patients met the inclusion criteria. Nine patients were excluded due to meeting exclusion criteria or failing quality control of B1 ctDNA quality control, resulting in a final cohort of 127 participants (Figure S1). The baseline clinical characteristics of all patients are summarized in Table S1 and were stratified according to baseline ctDNA status. Baseline ctDNA status was significantly related to primary tumor location (*p* < 0.01), stage (*p* = 0.03), and liver metastases (*p* < 0.01) (Table S1). Moreover, the baseline CA19‐9 levels in ctDNA‐positive patients were significantly greater than those in ctDNA‐negative patients (*p* = 0.007). We further analyzed the relationships between clinical features and baseline ctDNA levels and found that baseline ctDNA levels were higher in patients with M‐PDAC than in those with locally advanced PDAC (LA‐PDAC) (*p* < 0.001) (Figure 1A). Furthermore, ctDNA levels demonstrated significant elevation in two distinct clinical subgroups: patients with primary lesions localized to the pancreatic body/tail (*p* < 0.001) (Figure 1B) and those with liver metastases (*p* < 0.001) (Figure 1C). *p* values were adjusted for multiple comparisons using the Bonferroni method, with a corrected significance level of *α* = 0.0167 (0.05/3 tests). All associations remained statistically significant. Notably, no statistically significant correlation was observed between CA19‐9 positivity status and quantitative ctDNA levels (Figure 1D). Importantly, parallel analyses of maximum somatic allele frequency (MSAF) revealed concordant trends with ctDNA level across comparative cohorts (Figure 1E–H).

### 2.2 ctDNA Dynamics Indicate First‐Line Chemotherapy Efficacy

**FIGURE 2:**
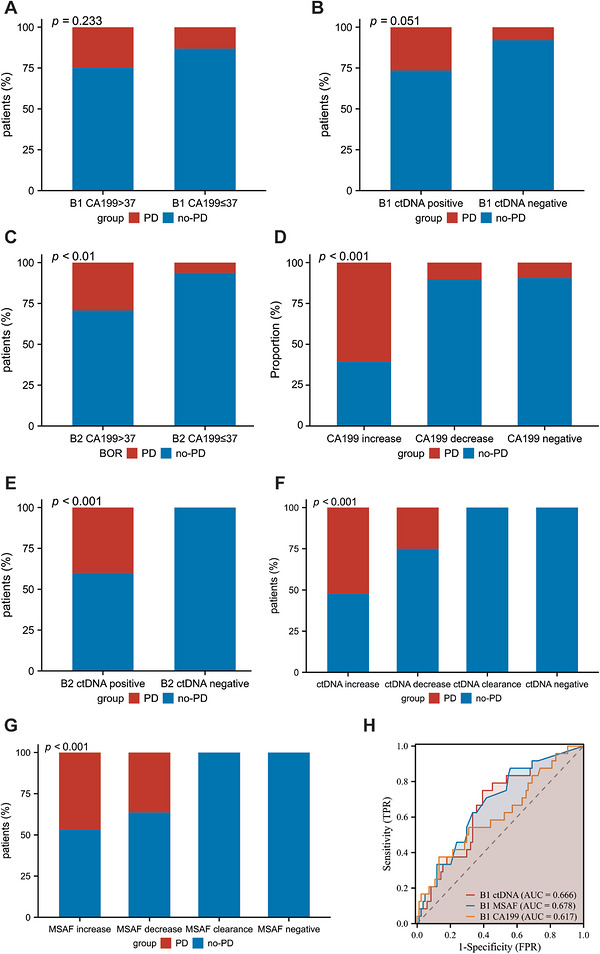
The role of ctDNA and CA19‐9 in assessing first‐line chemotherapy efficacy. (A, C, and D) Correlations between CA19‐9 status (A: B1 CA19‐9 status, C: B2 CA19‐9 status, D: CA19‐9 change) and therapeutic response to first‐line chemotherapy. (B, E, and F) Correlations between ctDNA status (B: B1 ctDNA status, E: B2 ctDNA status, F: ctDNA change) and therapeutic response to first‐line chemotherapy. (G) Correlations between changes in the MSAF and the therapeutic response to first‐line chemotherapy. (H) ROC curve analysis evaluating the predictive performance of the B1 ctDNA level, MSAF, and CA19‐9 for the optimal therapeutic response to first‐line chemotherapy.

In our cohort, the ctDNA detection rates significantly decreased from 80.0% (101/127) at B1 to 55.7% (49/88) at B2 (Figure 1I). A corresponding decrease in CA19‐9 positivity rates was observed, declining from 80.3% (102/127) to 70.4% (76/108) across matched timepoints (Figure 1J). Despite the limited number of ctDNA sampling timepoints, we assessed the correlation of CA19‐9 and ctDNA levels to reflect and predict disease progression. We initially focused on the associations between single‐time (B1 or B2) ctDNA and CA19‐9 positivity rates and the best overall response (BOR) to first‐line chemotherapy. B1 assessments revealed no statistically significant associations between CA19‐9 (*p* = 0.233) or ctDNA (*p* = 0.051) status and BOR (Figure 2A,B). Notably, B2 assessments revealed significant positive correlations between B2 CA19‐9 status (*p* < 0.01), B2 ctDNA status (*p* < 0.001), and BOR (Figure 2C,E). We then further investigated the dynamic changes in these indicators and their correlation with BOR. The results revealed that in patients with elevated CA19‐9 levels (Figure 2D), increased ctDNA levels (Figure 2F), and increased MSAF (Figure 2G), the proportion of patients with disease progression was significantly greater (*p* < 0.001). In contrast, in patients where these indicators remained negative or decreased, the BOR was either SD or partial response (PR). We subsequently plotted receiver operating characteristic (ROC) curves to assess the predictive efficacy of these indicators at B1 for BOR. The results revealed that ctDNA levels (AUC = 0.666; 95% CI 0.546–0.782) and the MSAF (AUC = 0.678; 95% CI 0.560–0.796) performed better than CA19‐9 levels (AUC = 0.617; 95% CI 0.484–0.751) (Figure 2H). On the basis of these findings, we observed that baseline single‐point ctDNA testing has limited clinical value. In contrast, dynamically monitoring changes in ctDNA levels and the MSAF more comprehensively reflects the response to first‐line chemotherapy.

### 2.3 Prognostic Value of Dynamic ctDNA Monitoring in PDAC

**FIGURE 3:**
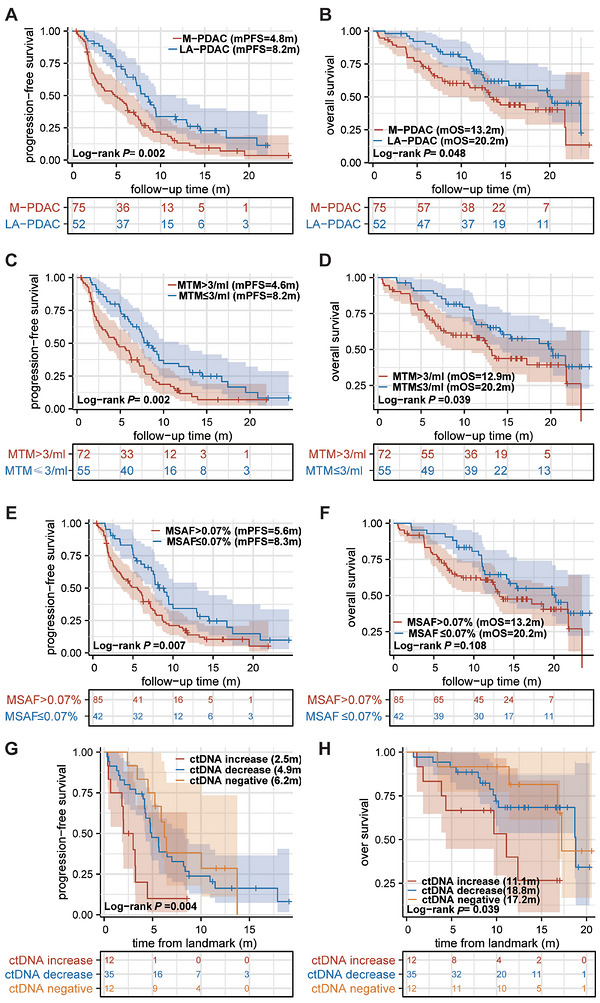
Prognostic impact of clinical characteristics and ctDNA on survival outcomes. (A and B) Impact of stage on PFS (A) and OS (B). (C and D) Impact of B1 ctDNA level on PFS (C) and OS (D) stratified by MTM 3/mL. (E and F) Impact of the B1 ctDNA MSAF on PFS (E) and OS (F) stratified by the MSAF 0.07%. (G and H) Impact of ctDNA changes on PFS (G) and OS (H) by landmark survival analysis.

To further investigate the prognostic value of ctDNA in PDAC, we analyzed the relationships among ctDNA levels, the MSAF, CA19‐9, and both progression‐free survival (PFS) and overall survival (OS). Consistent with previous clinical data, patients with LA‐PDAC exhibited significantly better PFS (8.2 vs. 4.8 months, *p* = 0.002, HR = 0.544, 95% CI 0.370–0.801) and OS (20.2 vs. 13.2 months, *p* = 0.048, HR = 0.603, 95% CI 0.369–0.985) than those with M‐PDAC (Figure 3A,B). Next, we analyzed the relationship between B1 ctDNA and survival. The results revealed that PDAC patients with B1 ctDNA levels greater than 3 MTM/mL had a significantly shorter median PFS (4.6 vs. 8.2 months, *p* = 0.002, HR = 0.541, 95% CI 0.367–0.798) than those with 3 MTM/mL or less (Figure 3C). Similarly, patients with B1 ctDNA MSAF greater than 0.07% had a significantly shorter median PFS (5.6 vs. 8.3 months, *p* = 0.007, HR = 0.566, 95% CI 0.383–0.835) than those with an MSAF of 0.07% or less (Figure 3E). In terms of OS, B1 ctDNA levels and the MSAF exhibited similar trends, with patients whose ctDNA levels were greater than 3 MTM/mL (12.9 vs. 20.2 months, *p* = 0.039, HR = 0.596, 95% CI 0.365–0.972) and whose MSAF was greater than 0.07% (13.2 vs. 20.2 months, *p* = 0.108, HR = 0.654, 95% CI 0.398–1.077) showing shorter OS (Figure 3D,F). The B1 ctDNA status did not significantly correlate with PFS or OS (Figure S2A,B). To validate ctDNA level (> 3 MTM/mL) and MSAF (> 0.07%) as reliable prognostic biomarkers, we analyzed an external validation set (*n* = 62) (Figure S3). Concordant with our primary cohort, levels exceeding these thresholds were significantly associated with shorter PFS (Figure S3A,C), while no statistically significant difference was observed for OS (Figure S3B,D). Next, we focused on whether dynamic changes in ctDNA could predict the prognosis of PDAC patients. The results revealed that, compared with a decrease or sustained negativity in ctDNA levels, an increase in ctDNA levels was significantly associated with a shorter PFS (*p* = 0.004; Figure 3G) and OS (*p* = 0.039; Figure 3H) by landmark survival analysis.

To evaluate the impact of clinical characteristics, CA19‐9, and ctDNA on survival, we conducted both univariate and multivariate Cox regression analyses for PFS (Table 1) and OS (Table 2). For PFS, univariate Cox regression analysis indicated that N2 (HR = 2.320, 95% CI 1.034–5.202, *p* = 0.041), stage (HR = 1.855, 95% CI 1.240–2.776, *p* = 0.003), liver metastasis (HR = 1.866, 95% CI 1.263–2.756, *p* = 0.002), B1 CA19‐9 levels (*p* = 0.002), B1 ctDNA MSAF (HR = 13.224, 95% CI 3.231–54.114, *p* < 0.001), longitudinal MSAF (HR = 29.797, 95% CI 4.492–197.661, *p* < 0.001), longitudinal ctDNA status (HR = 1.549, 95% CI 1.022–2.349, *p* = 0.039), and longitudinal CA19‐9 level (*p* = 0.001) were significantly associated with PFS. Multivariate Cox regression analysis revealed that N1 (HR = 1.657, 95% CI 1.109–2.474, *p* = 0.014), N2 (HR = 1.792, 95% CI 1.071–2.999, *p* = 0.026), stage (HR = 1.739, 95% CI 1.197–2.527, *p* = 0.004), longitudinal MSAF (HR = 7.442, 95% CI 2.167–25.551, *p* = 0.001), and longitudinal CA19‐9 level (*p* < 0.001) was significantly associated with PFS (Table 1). In terms of OS, univariate Cox analyses suggested that age (HR = 1.051, 95% CI 1.020–1.083, *p* = 0.001), N2 (HR = 3.210, 95% CI 1.272–8.104, p = 0.014), liver metastasis (HR = 1.939, 95% CI 1.179–3.191, p = 0.009), B1 CA19‐9 levels (*p* = 0.002), B1 ctDNA MSAF (HR = 8.620, 95% CI 1.449–51.274, *p* = 0.018), longitudinal MSAF (HR = 129.102, 95% CI 14.332–1162.932, *p* < 0.001), longitudinal ctDNA status (HR = 2.054, 95% CI 1.173–3.597, *p* = 0.012), and longitudinal CA19‐9 level (*p* = 0.002) were significantly associated with OS. However, multivariate Cox regression analysis revealed that age (HR = 1.067, 95% CI 1.046–1.089, *p* < 0.001), N2 (HR = 3.631, 95% CI 2.072–6.363, *p* < 0.001), longitudinal MSAF (HR = 10.451, 95% CI 2.731–39.999, *p* = 0.001), and longitudinal CA19‐9 level (*p* < 0.001) was significantly associated with OS (Table 2).

**TABLE 1:** Univariate and multivariate Cox regression analyses for PFS.

Characteristics	Total (*N*)	Univariate analysis		Multivariate analysis	
		HR (95% CI)	*p* value	HR (95% CI)	*p* value
Age	127	1.014 (0.992–1.036)	0.225		
Sex	127				
F	57	Reference			
M	70	1.011 (0.685–1.492)	0.955		
T stage	127				
T1‐2	41	Reference			
T3	27	0.803 (0.461–1.397)	0.437		
T4	59	1.001 (0.646–1.551)	0.997		
N stage	127				
N0	24	Reference		Reference	
N1	18	1.799 (0.922–3.510)	0.085	1.657 (1.109–2.474)	**0.014**
N2	10	2.320 (1.034–5.202)	**0.041**	1.792 (1.071–2.999)	**0.026**
Nx	75	1.208 (0.711–2.052)	0.484	1.042 (0.741–1.466)	0.811
Stage	127				
Locally advanced	52	Reference		Reference	
Metastatic	75	1.855 (1.240–2.776)	**0.003**	1.739 (1.197–2.527)	**0.004**
Liver metastases	127				
No	71	Reference		Reference	
Yes	56	1.866 (1.263–2.756)	**0.002**	0.991 (0.689–1.426)	0.962
ECOG	127				
0	63	Reference			
1–2	64	1.085 (0.734–1.604)	0.683		
B1 CA19‐9 status	127				
B1 CA19‐9 ≤ 37	25	Reference			
B1 CA19‐9 > 37	102	1.089 (0.660–1.795)	0.740		
B1 CA19‐9 level	127	1.000 (1.000–1.000)	**0.002**		
B1 ctDNA status	127				
B1 ctDNA‐negative	27	Reference			
B1 ctDNA‐positive	100	1.376 (0.843–2.248)	0.202		
B1 ctDNA level	127	1.000 (1.000–1.000)	0.609		
B1 ctDNA MSAF	127	13.224 (3.231–54.114)	**< 0.001**		
Longitudinal MSAF	127	29.797 (4.492–197.661)	**< 0.001**	7.442 (2.167–25.551)	**0.001**
Longitudinal ctDNA level	127	1.000 (1.000–1.000)	0.689		
Longitudinal ctDNA status	127	1.549 (1.022–2.349)	**0.039**		
Longitudinal CA19‐9 level	127	1.000 (1.000–1.000)	**0.001**	1.000 (1.000–1.000)	**< 0.001**
Longitudinal CA19‐9 status	127	1.150 (0.717–1.846)	0.562		

^a^ Time‐dependent Cox regression was used for longitudinal biomarkers, including ctDNA and CA19‐9, to minimize guarantee‐time bias.

^b^ To avoid potential multicollinearity, B1 CA19‐9 level, B1 ctDNA MSAF, and longitudinal ctDNA status—despite being significantly associated with outcome in univariate analysis (*p* < 0.05)—were excluded from the multivariate model.

Bold values indicate statistically significant results (*p* < 0.05).

**TABLE 2:** Univariate and multivariate Cox regression analyses for OS.

Characteristics	Total (*N*)	Univariate analysis		Multivariate analysis	
		HR (95% CI)	*p* value	HR (95% CI)	*p* value
Age	127	1.051 (1.020–1.083)	**0.001**	1.067 (1.046–1.089)	**< 0.001**
Sex	127				
F	57	Reference			
M	70	1.270 (0.772–2.088)	0.346		
T stage	127				
T1‐2	41	Reference			
T3	27	0.855 (0.410–1.781)	0.675		
T4	59	1.221 (0.702–2.122)	0.479		
N stage	127				
N0	24	Reference		Reference	
N1	18	1.643 (0.667–4.049)	0.281	1.352 (0.781–2.339)	0.281
N2	10	3.210 (1.272–8.104)	**0.014**	3.631 (2.072–6.363)	**< 0.001**
Nx	75	1.419 (0.713–2.822)	0.319	1.080 (0.703–1.659)	0.726
Stage	127				
Locally advanced	52	Reference			
Metastases	75	1.671 (0.998–2.798)	0.051		
Liver metastases	127				
No	71	Reference		Reference	
Yes	56	1.939 (1.179–3.191)	**0.009**	1.208 (0.869–1.679)	0.262
ECOG	127				
0	63	Reference			
1–2	64	1.502 (0.910–2.481)	0.112		
B1 CA19‐9 status	127				
≤ 37	25	Reference			
> 37	102	1.358 (0.704–2.620)	0.362		
B1 CA19‐9 level	127	1.000 (1.000–1.000)	**0.002**		
B1 ctDNA status	127				
Negative	27	Reference			
Positive	100	1.168 (0.645–2.114)	0.608		
B1 ctDNA level	127	1.000 (1.000–1.000)	0.704		
B1 ctDNA MSAF	127	8.620 (1.449–51.274)	**0.018**		
Longitudinal MSAF	127	129.102 (14.332–1162.932)	**< 0.001**	10.451 (2.731–39.999)	**0.001**
Longitudinal ctDNA level	127	1.000 (1.000–1.000)	0.255		
Longitudinal ctDNA status	127	2.054 (1.173–3.597)	**0.012**		
Longitudinal CA19‐9 level	127	1.000 (1.000–1.000)	**0.002**	1.000 (1.000–1.000)	**< 0.001**
Longitudinal CA19‐9 status	127	1.408 (0.760–2.608)	0.277		

^a^ Time‐dependent Cox regression was used for longitudinal biomarkers, including ctDNA and CA19‐9, to minimize guarantee‐time bias.

^b^ To avoid potential multicollinearity, B1 CA19‐9 level, B1 ctDNA MSAF, and longitudinal ctDNA status—despite being significantly associated with outcome in univariate analysis (*p* < 0.05)—were excluded from the multivariate model.

Bold values indicate statistically significant results (*p* < 0.05).

### 2.4 Clinical Utility of ctDNA in CA19‐9‐Negative PDAC Patients

**FIGURE 4:**
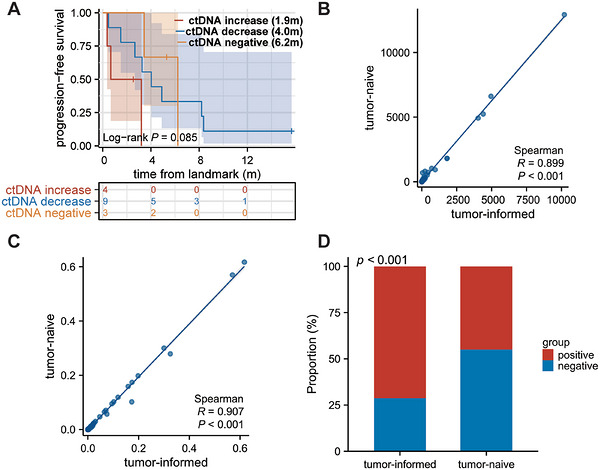
Clinical utility of ctDNA in CA19‐9‐negative cohort and comparison of tumor‐naive versus tumor‐informed ctDNA assays. (A) Impact of ctDNA changes on PFS in the CA19‐9‐negative cohort by landmark survival analysis. (B and C) Concordance of ctDNA level (B) and MSAF (C) between the tumor‐naive and tumor‐informed ctDNA assays. (D) Comparison of positivity rates in tumor‐naive versus tumor‐informed ctDNA assays.

The clinical value of CA19‐9 levels and their dynamic changes in PDAC patients has been widely studied (Figure S4). Our cohort also reaffirmed that an elevated B1 CA19‐9 level correlated with shorter PFS (Figure S4E) and OS (Figure S4F). Furthermore, we focused on PDAC patients who were CA19‐9 negative and investigated the clinical utility of ctDNA in this group. The results revealed that using the median B1 ctDNA level as the cut‐off did not significantly predict the prognosis of these patients (Figure S5A,B). Furthermore, the B1 ctDNA status was not significantly associated with PFS and OS following first‐line chemotherapy (Figure S5C,D). Focusing on the relationship between dynamic changes in ctDNA and BOR, the results revealed that patients with increased ctDNA levels had a higher proportion of progressive disease (PD) (Figure S5F). The results of the K–M survival curve showed that the PFS of patients with increased ctDNA levels was shorter than that of patients with decreased or sustained negative ctDNA levels (Figure 4A). However, there were no significant differences among the three groups in terms of OS (Figure S5E). This study included a total of 25 CA19‐9‐negative PDAC patients at baseline, with 20 patients for whom both B1 and B2 ctDNA tests were available. The sample size was relatively small. Future studies with larger cohorts are needed to validate the application of ctDNA in CA19‐9‐negative PDAC patients.

### 2.5 Comparison of Tumor‐Naive Versus Tumor‐Informed ctDNA Assays

In our cohort, tumor tissue NGS results were available for 45 patients prior to B1 ctDNA testing; therefore, a tumor‐informed ctDNA assay was applied for these 45 patients, whereas the remaining 82 patients were analyzed using a tumor‐naive assay. To enable a quantitative comparison between the two ctDNA detection strategies, samples analyzed using the tumor‐informed approach (45 cases at B1 and 35 cases at B2) were recalculated using the tumor‐naive algorithm based on the same sequencing data. Spearman correlation analysis was subsequently performed to evaluate the consistency of ctDNA‐related metrics between the two methods. The results demonstrated a strong positive correlation between the two detection methods for ctDNA levels (Figure 4B) (*R* = 0.899) and MSAF (Figure 4C) (*R* = 0.907), both of which were statistically significant (*p* < 0.001). After converting all tumor‐informed results to tumor‐naive–derived metrics, K–M survival analyses were repeated (Figure S6). In line with the findings of the preceding analyses, the results demonstrated that a ctDNA level greater than 3 MTM/mL (*p* = 0.006) (Figure S6A) and an MSAF greater than 0.07% (*p* = 0.018) (Figure S6C) were both significantly associated with shorter PFS.

Further comparison of ctDNA positivity rates revealed that the tumor‐informed approach achieved a significantly higher detection rate than the tumor‐naive method (*p* < 0.001) (Figure 4D). These findings underscore the clinical value of comprehensive tumor tissue NGS profiling and suggest that, in patients with PDAC, optimizing tissue‐based genomic characterization whenever feasible may substantially enhance the sensitivity of ctDNA detection.

### 2.6 Genomic Features of PDAC Based on ctDNA

**FIGURE 5:**
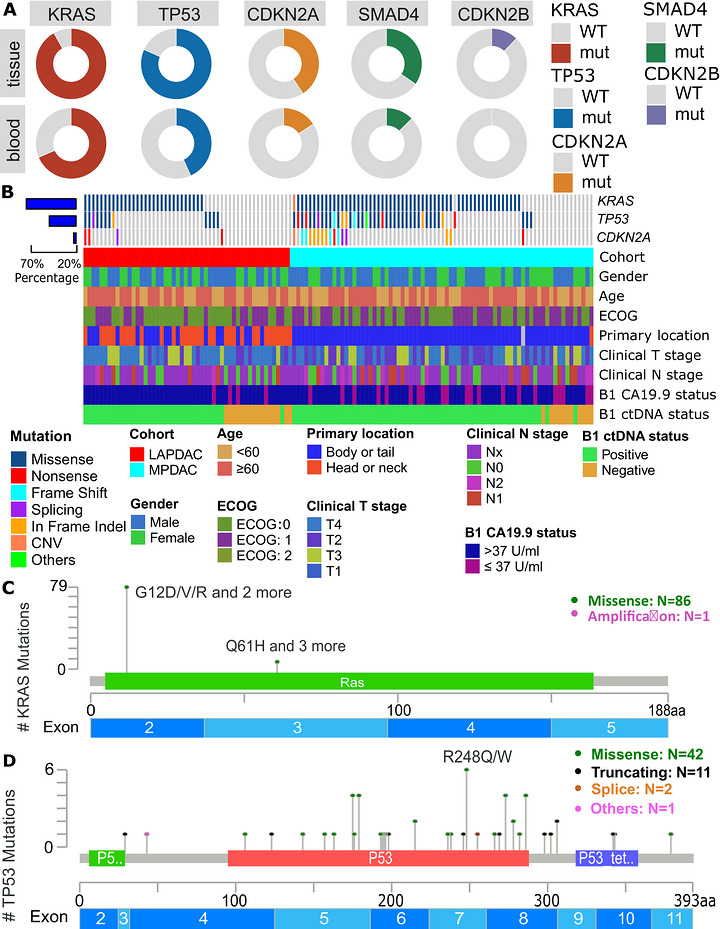
Genomic features of PDAC samples tested via ctDNA. (A) Comparative detection rates of 5‐core genes in tissue NGS and ctDNA profiling. (B) Mutation profiles of the top 3 mutated genes at baseline in this study. (C and D) Distribution of KRAS (C) and TP53 (D) mutation sites.

We explored the detection rates of 5‐core genes with high frequency in PDAC patients via both tumor tissue sample NGS testing and ctDNA testing. The mutation frequencies in blood samples were much lower than those in tissue samples (*KRAS* 91.84% vs. 67.7%, *p* < 0.01; *TP53* 87.76% vs. 43.31%, *p* < 0.001; *CDKN2A* 42.86% vs. 15.75%, *p* < 0.001; *SMAD4* 38.78% vs. 12.59%, *p* < 0.001; *CDKN2B* 42.86% vs. 17.32%, *p* < 0.001), which was reasonable due to the high false negative rate of ctDNA detection, especially in LA‐PDAC (Figure 5A). We then profiled the genomic features of PDAC patients and investigated their relationship with survival. As illustrated in Figure 5B, the genes most frequently mutated in our study included *KRAS*, *TP53*, and *CDKN2A*. The prognostic impacts of *KRAS* (Figure S7A), *TP53* (Figure S7B), and *CDKN2A* (Figure S7C) signatures on both PFS and OS are comprehensively illustrated in Figure S7. In addition, the distribution of mutation sites for *KRAS* and *TP53* is shown in Figure 5C,D. The majority (90.7%) of *KRAS* mutations were concentrated on the G12 site, including G12D (52.3%), G12V (23.3%), G12R (10.5%), and G12C (3.5%) (Figure 5C). In contrast, no hotspot mutation for *TP53* was identified, and most of the mutations were dispersed across the p53 DNA‐binding domain (Figure 5D). Missense mutations accounted for 75% of all *TP53* mutations, and virtually all of them were located within the p53 DNA‐binding domain.

### 2.7 ctDNA Dynamics Indicate the Progression of PDAC

**FIGURE 6:**
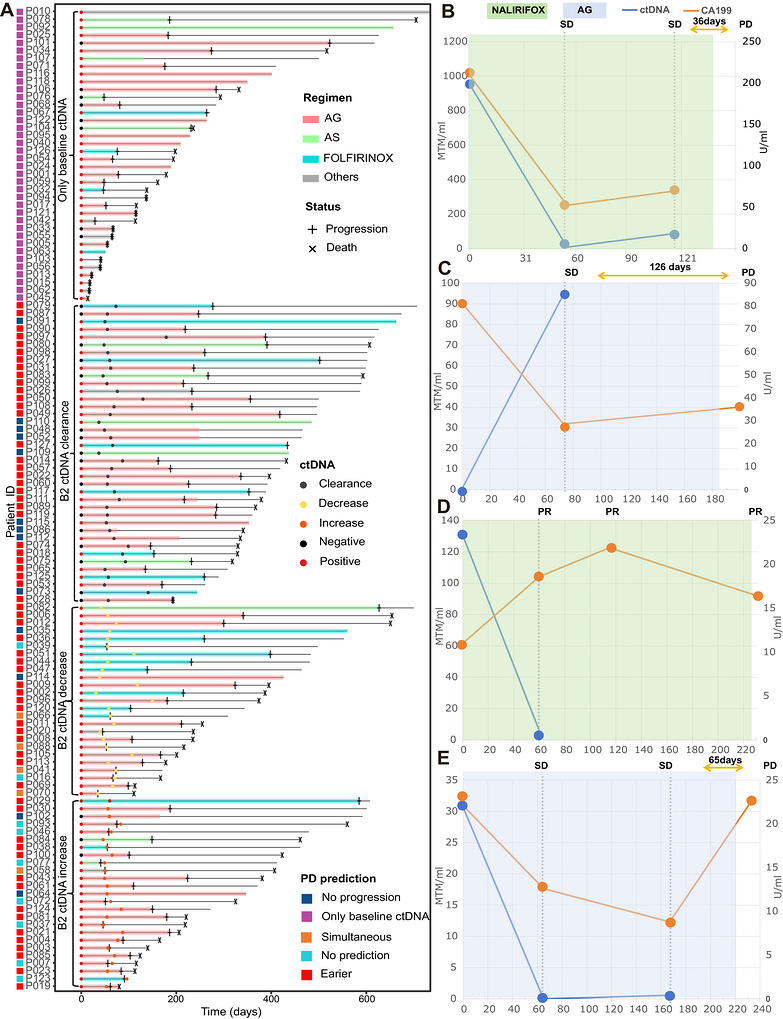
Longitudinal ctDNA dynamics as a predictive biomarker for first‐line therapeutic response. (A) Swimmer plot showing ctDNA dynamics and disease progression. (B–E) Illustrations of representative cases indicating ctDNA dynamics in predicting disease progression or response. AG, nab‐paclitaxel + gemcitabine; FOLFIRINOX, leucovorin + 5‐FU + oxaliplatin + irinotecan; NALIRIFOX: leucovorin + 5‐FU + oxaliplatin + liposomal irinotecan; PD, progressive disease; PR, partial response; SD, stable disease.

We assessed the capacity of ctDNA dynamics to reflect and predict disease progression (Figure 6A). Among the 127 patients undergoing longitudinal ctDNA monitoring, 25 cases (19.7%) demonstrated rising ctDNA levels, 24 (18.9%) exhibited declining ctDNA titers, 23 (18.1%) achieved molecular clearance (defined as sustained conversion from ctDNA‐positive to ctDNA‐negative status), and 16 (12.6%) maintained undetectable ctDNA throughout surveillance. Among the patients whose ctDNA indicated progression, 21 patients (91.3%) had concordant imaging evidence of progression, and 19 patients (82.6%) demonstrated elevated ctDNA levels before imaging confirmed disease progression, with a median lead time of 92 days (range: 1–585 days). Representative cases were depicted in Figure 6B–E, for which ctDNA predicted progression 36–126 days prior to radiological examinations.

## 3 Discussion

PDAC is the most prevalent pathological type of pancreatic cancer. The majority of patients are diagnosed with advanced cancer, including locally advanced and metastaticPDAC, making them ineligible for curative surgery. The overall 5‐year survival rate is less than 11% [39]. This poses significant challenges in effectively evaluating treatment efficacy and predicting patient survival in clinical practice. This study focused on the application of ctDNA in advanced‐stage PDAC, explored its potential in predicting survival, assessing treatment efficacy, and guiding therapy in these patients.

First, we focused on the prognostic value of ctDNA. A meta‐analysis comprising 25 clinical studies and 2326 patients demonstrated that higher concentrations of ctDNA are associated with shorter OS and PFS [40]. In a study of 104 PDAC patients, the median OS was 6.5 months for those with detectable ctDNA, whereas it was 19 months for those without detectable ctDNA [41]. Our study reached similar conclusions. On the basis of the results from the 127 PDAC patients in this study, ctDNA levels above 3 MTM/mL and MSAFs exceeding 0.07% at B1 were associated with shorter PFS and OS (Figure 3C–F). In addition, this study aimed to address two other clinical questions: (1) The first question is whether ctDNA can serve as an early predictor of treatment response in patients with PDAC. Figure 2F,G demonstrates that a decrease in or clearance of ctDNA and MSAF generally indicates disease control, whereas an increase may suggest disease progression. In addition, Figure 3G shows that elevated ctDNA levels are associated with shorter PFS. In three cases where patients initially exhibited SD during their first efficacy evaluation, ctDNA indicated disease progression 36, 126, and 65 days earlier than imaging (Figure 6B,C,E). A study published in 2017 corroborated our findings [41]. That trial included 102 patients with advanced PDAC and reported that OS was significantly shorter in the ctDNA‐positive group than in the ctDNA‐negative group (6.5 vs. 19.0 months; *p* < 0.001) [41]. Currently, there are no large‐scale studies in the field of PDAC, such as the DYNAMIC study in lung cancer, that accurately predict the lead time of ctDNA over imaging through longitudinal monitoring [42]. In 2019, Groot's research team demonstrated that in localized PDAC, ctDNA could detect disease recurrence in 90% of patients (27 out of 30), approximately 84 days earlier than radiographic imaging [43]. On the basis of the results of our study, ctDNA can be used to detect disease progression approximately 1 month before imaging can be used. (2) The second clinical question we sought to address was whether ctDNA can serve as a reliable biomarker for clinical guidance in CA19‐9‐negative PDAC patients. This study places special emphasis on CA19‐9‐negative patients. The baseline analysis revealed no statistically significant differences in ctDNA levels or MSAFs between the CA19‐9‐positive (> 37 U/mL) and CA19‐9‐negative (≤ 37 U/mL) groups (Figure 1D,H). Owing to the relatively small sample size of CA19‐9‐negative patients (*n* = 25), baseline ctDNA status and levels were not effective predictors of PFS or OS (Figure S5A‒D). However, focusing on dynamic changes in ctDNA, we found that an increase in ctDNA was associated with shorter PFS (Figure 4A). On the basis of these findings, we believe that dynamic monitoring of ctDNA in CA19‐9‐negative patients can provide valuable prognostic information and aid in clinical decision‐making.

In addition, we assessed the value of ctDNA in the context of precision medicine. Obtaining sufficient tissue for NGS in some patients with PDAC is challenging. This is often due to the reliance on fine‐needle biopsy for diagnosis, which yields limited tissue samples insufficient for NGS. Based on sequencing data, we applied an algorithm to re‐analyze the “tumor‐informed” cohort based on the methodology used for the “tumor‐naive” cohort. The results from both approaches were highly consistent, with a higher detection rate observed in the “tumor‐informed” cohort (Figure 4D). Therefore, integrating tumor‐informed and tumor‐naive strategies allows a larger proportion of patients to benefit from ctDNA‐based monitoring while maintaining clinically meaningful predictive performance. Furthermore, repeated biopsies are often not feasible when PDAC patients experience disease progression. Comparing the detection rates of the 5‐core genes in the tissue and peripheral blood samples, we found that the mutation detection rate in the tissue samples was greater than that in the ctDNA samples (Figure 5A). This finding indicates that ctDNA cannot replace tissue‐based NGS with current detection methods.

KRAS is one of the most prevalent driver genes in PDAC and is present in more than 90% of cases. Other commonly identified driver genes include TP53, SMAD4, and CDKN2A [44]. In this study, the detection rate of KRAS in ctDNA was 67.7%, which is lower than the 91.84% observed in tissue samples but aligns with literature reports ranging from 62.5% to 95% [44]. Previous studies have focused predominantly on the KRAS gene in ctDNA [44, 45]. The PRINCE phase 2 chemoimmunotherapy trial included 129 first‐line PDAC patients. Using highly sensitive pre‐amplification ddPCR, ctKRAS mutations were detected in 86 out of 115 baseline plasma samples (74.8%) collected before the first cycle, the first day (C1D1), of treatment. The ctKRAS G12D mutation (log‐rank *p*  =  0.0010) was significantly associated with shorter OS, whereas the ctKRAS G12V mutation (*p*  =  0.7101) was not significantly correlated with OS. Similar conclusions were reached in a study of 85 patients receiving standard first‐line chemotherapy [46]. In this study, no significant differences in survival were detected among patients with the KRAS G12D, G12V, and G12R mutations detected in ctDNA (Figure S4A,B). In contrast, when comparing the impact of two other frequently mutated genes, CDKN2A and TP53, baseline detection of TP53 mutations in ctDNA was associated with shorter PFS (Figure S4E).

Tumor metastasis is a multistep process involving local invasion, intravasation, dissemination through the bloodstream, and colonization at distant sites [21, 47]. Epithelial‐mesenchymal transition of tumor cells, CTCs, and the secretion of chemokines all play promotive roles in this process [48]. Based on these biological mechanisms, the higher ctDNA positivity rate observed in M‐PDAC can be further explained (Table S1). Combining the traditional tumor marker CA19‐9 with DNA methylation markers and microRNAs (such as miR‐21, miR‐155, and miR‐196a) has been shown to improve the sensitivity and specificity of PDAC diagnosis [49, 50]. Moreover, multi‐omics‐based predictive models integrating genomic, proteomic, transcriptomic, and metabolomic data can more comprehensively elucidate the molecular drivers of PDAC, offering promise for future precision stratification and treatment [21, 51]. Meanwhile, we also acknowledge the current limitations of ctDNA in clinical practice: clinical decision‐making still primarily relies on imaging and tissue‐based NGS, and ctDNA has not yet been established as an independent biomarker to guide clinical decisions in PDAC [52].

This study has certain limitations. First, the study's sample size was relatively small, particularly in the CA19‐9 negative subgroup. Although the hazard ratios demonstrate statistical significance (*p* < 0.05) and 95% CI consistently below 1, the relative width of the CI reflects limitations related to sample size, event numbers, and PDAC heterogeneity. To confirm the reliability of these findings, we plan to further conduct multi‐center clinical studies with larger sample sizes to independently validate the proposed ctDNA cutoffs and to confirm the reproducibility and clinical utility of ctDNA detection and its dynamic changes in PDAC. Second, not all patients had tissue samples available for NGS testing, which may have led to some mutations being undetected owing to panel coverage limitations. Third, our original plan involved intensive blood sampling to accurately determine the lead time of ctDNA over imaging. However, owing to the generally poor condition of PDAC patients, intensive blood sampling is challenging. Consequently, we revised the protocol to collect blood samples at two key points: before the initiation of treatment (baseline, B1) and at the first response assessment (B2). Finally, peripheral blood ctDNA testing may yield false‐negative results, particularly in cases of LA‐PDAC. Therefore, exercising caution when making treatment decisions on the basis of these test outcomes is crucial.

## 4 Conclusion

On the basis of dynamic ctDNA monitoring of 127 patients with advanced PDAC, this study demonstrated that ctDNA levels above 3 MTM/mL and MSAF values exceeding 0.07% are indicators of poor prognosis. Conversely, a decrease in or clearance of ctDNA and the MSAF suggest better disease control. In addition, in CA19‐9‐negative patients, dynamic ctDNA monitoring was able to indicate disease control and predict patient prognosis.

## 5 Materials and Methods

### 5.1 Patients and Samples

This observational, prospective cohort study recruited treatment‐naive patients with locally advanced or metastaticPDAC who were admitted to the Department of Medical Oncology at Peking Union Medical College Hospital to receive first‐line systematic treatment (Beijing, China). All treatment decisions were made by the physicians in accordance with current clinical guidelines and patient preferences, independent of the study protocol. The inclusion criteria were as follows: (1) histologically confirmed advanced PDAC (locally advanced or metastatic); (2) aged 18–70 years with no sex‐based restrictions; (3) Eastern Cooperative Oncology Group (ECOG) performance status ≤ 2; (4) life expectancy ≥ 3 months; and (5) voluntary participation with written informed consent. The exclusion criteria were as follows: (1) history of other malignancies within 5 years; (2) prior anti‐cancer therapy within 6 months; (3) clinically significant comorbidities that may compromise study completion or life expectancy (e.g., psychiatric disorders or uncontrolled hypertension).

Approximately 20 mL peripheral blood samples were collected longitudinally at two time points: (1) prior to the initiation of first‐line chemotherapy (baseline, B1) and (2) during the first response assessment (B2) via Streck tubes (CWBIO, Taizhou, China). At the first efficacy evaluation, serum CA19‐9 and radiographic imaging were performed. Tumor response was evaluated according to the RECIST version 1.1 criteria [7]. Patients who failed to yield valid ctDNA data at the B2 timepoint were not subjected to data imputation. Instead, they were designated as having “missing data” and consequently excluded from any relevant dynamic or survival analyses. PFS was defined as the time from the start of treatment to disease progression or tumor‐related death. OS was defined as the time from the start of treatment (or for patients who died prior to receiving treatment, from the first blood draw) to all‐cause mortality.

The study was conducted in accordance with the Declaration of Helsinki and approved by the Ethics Committee of Peking Union Medical College Hospital (Approval No. I‐23PJ491). This study was registered with ClinicalTrials.gov (NCT05802420 for the metastatic cohort and NCT05802394 for the locally advanced cohort). All patients were followed via outpatient visits or telephone interviews, with the last follow‐up date recorded as March 2025. The ctDNA and clinical data for the external validation set were derived from two previously published clinical studies [33, 53].

### 5.2 Approaches for ctDNA Detection

By leveraging the specific mutation profile of tumor tissue, tumor‐informed ctDNA detection effectively minimizes false‐positive results arising from the interference of background noise and multiple hypothesis testing, thereby outperforming tumor‐naive strategies [54, 55]. In this study, we applied two different approaches for ctDNA detection. For patients who had undergone NGS of tumor tissue, tumor‐informed personalized ctDNA detection was conducted. For patients without sequencing of tumor tissue, tumor‐naive ctDNA detection methods, including KRAS, CDKN2A, TP53, PIK3CA, and BRAF, were used. Both tumor‐informed and tumor‐naive ctDNA detection methods achieve sequencing depths exceeding 100,000×.

### 5.3 Sequencing and Bioinformatics Analyses

ctDNA detection comprises several key steps, including plasma separation, DNA extraction, library construction, probe synthesis, hybridization, sequencing, and bioinformatics analyses, which have been described previously [56, 57]. Briefly, peripheral blood samples were first subjected to two‐step centrifugation for the segregation of plasma from lymphocytes. Circulating‐free DNA (cfDNA) was extracted from plasma via a MagMAX Cell‐Free DNA Isolation Kit (Thermo Fisher, Waltham, MA, USA). DNA libraries were constructed via a KAPA DNA Library Preparation Kit (Kapa Biosystems, Wilmington, MA, USA). For patients with available tumor mutation profiles, the top‐ranked 20 mutations (excluding copy number variants [CNVs]) were selected to synthesize the tumor‐specific customized panel, which was combined with the 5‐gene core panel for ctDNA detection. Otherwise, a 5‐gene core panel was employed directly for ctDNA sequencing. Targeted sequencing was performed with a Gene+seq2000 sequencer (Geneplus, Suzhou, China).

After removing terminal adaptor sequences and low‐quality reads, the Burrows–Wheeler Aligner (BWA; version 0.7.12‐r1039) was used to align the clean reads to the reference human genome (hg19). Single nucleotide variants (SNVs), small insertions and deletions (InDels), CNVs, and structural variants (SVs) were identified via MuTect (version 1.1.4)/NChot, GATK (version 3.4–46‐gbc02625), and CONTRA (version 2.0.8), respectively. All variants were verified via the Integrative Genomics Viewer (IGV). For a given sample, the ctDNA level, expressed as the mean number of tumor molecules per milliliter (MTM/mL), was quantified as the concentration of cfDNA multiplied by the mean variant allele frequency of the sample, multiplied by 2000, and finally divided by 3.3. MSAF was used to estimate the ctDNA fraction in plasma. The intra‐run (repeatability) and inter‐run (reproducibility) precision of the tumor‐informed assay have been previously validated in a previous study [58]. This validation demonstrated a high detection concordance rate of 100%.

### 5.4 Statistical Analysis

ctDNA levels exhibited a right‐skewed distribution (Shapiro‒Wilk *p* < 0.001) (Figure S8). Logarithmic transformation (base 2) was applied after adding a constant (+1) to accommodate zero values. Continuous parameter comparisons between two groups were conducted via Student's t test or the Mann‒Whitney U test. The non‐parametric Spearman correlation coefficient was computed to assess the correlation between two continuous variables. The relationships between categorical variables were examined via the chi‐square test or Fisher's exact test. Survival analyses were performed via the R packages survival (v3.5.5) and survminer (v0.4.9). The optimal thresholds for ctDNA levels (>3 MTM/mL) and MSAF (>0.07%) were determined using the surv_cutpoint function from the survminer package, which identifies the optimal cut‐off by maximally separating survival outcomes. Specifically, Kaplan‒Meier survival curves for PFS and OS were drawn, and the log‐rank test was used to compare survival between different groups. Landmark survival analysis was applied to the time‐varying covariates. The first efficacy evaluation for most patients occurred at 2–3 months posttreatment, with the landmark set at 90 days after B1. Enrollment in the landmark survival analysis was restricted to patients who had surpassed this 90‐day landmark without disease progression or death, and who had completed both scheduled ctDNA assessments (B1 and B2). When survival among three or more groups was compared, Benjamini‒Hochberg (BH) correction was used for pairwise post hoc analyses of survival differences. In addition, univariate and multivariate Cox regression analyses were performed to investigate the factors that may influence survival. Variables that were significant in univariate analysis (*p* < 0.05), including B1 CA19‐9 level, B1 ctDNA MSAF, and longitudinal ctDNA status, were withheld from the final multivariate Cox model to avoid collinearity with the longitudinal metrics.

Unless otherwise specified, *p* < 0.05 was considered statistically significant. The mutation profile was drawn via the R package ComplexHeatmap (v2.14.0), and lollipop plots were generated via an online tool (https://www.cbioportal.org/mutation_mapper). Time‒dependent ROC curves were drawn via the R package timeROC (v0.4). All figures were generated via the ggplot2 R package (v3.4.4). Analyses were performed using the R statistical software version 4.2.1.

## Author Contributions

T.Y. and H.T. conceived and designed the study. T.Y., H.T., J.Y., and M.Y. performed the literature search. T.Y., H.T., M.Y., and R.C. analyzed the data. T.Y. generated the figures and tables and wrote the manuscript. C.B. critically reviewed the manuscript. C.B., Y.W., and Y.C. supervised the research. All authors have read and approved the final manuscript.

## Funding

This study was supported by the Chinese Academy of Medical Sciences Innovation Fund for Medical Sciences (2023‐I2M‐2‐002), the National High Level Hospital Clinical Research Funding (2025‐PUMCH‐A‐138), and the Peking Union Medical College Hospital Research Funding for Postdoc (kyfyjj202508).

## Ethics Statement

Informed consent was obtained from all participants in the study. The study was conducted in accordance with the Declaration of Helsinki and approved by the Ethics Committee of Peking Union Medical College Hospital (Approval No. I‐23PJ491). This study was registered with ClinicalTrials.gov (NCT05802420 for the metastatic cohort and NCT05802394 for the locally advanced cohort). Both trials were prospectively registered prior to patient recruitment.

## Conflicts of Interest

Mingming Yuan and Rongrong Chen are employees of Geneplus‐Beijing. The other authors declare no conflicts of interest.

## Supporting information




**Figure S1**: Study flow chart. PDAC, pancreatic ductal adenocarcinoma.
**Figure S2**: Impact of ctDNA status on PFS and OS.
**Figure S3**: Prognostic impact of ctDNA on survival outcomes in external validation cohort.
**Figure S4**:Impact of clinical characteristics and CA19‐9 on PFS and OS.
**Figure S5**:Clinical utility of ctDNA in CA19‐9‐negative PDAC patients.
**Figure S6**:Impact of B1 ctDNA on PFS and OS by tumor‐naive ctDNA assays.
**Figure S7**. Prognostic significance of ctDNA‐identified somatic mutations.
**Figure S8**:Assessment of normality for ctDNA level and log2 (count+1) using Q‒Q (A) and P‐P (B) plots.
**Table S1**: Baseline characteristics stratified according to ctDNA status.

## Data Availability

The data that support the findings of this study are available from the corresponding author upon reasonable request.
