# Supplementary material for: Dynamics of Circulating Tumor DNA (ctDNA) Predict Treatment Efficacy and Prognosis in Patients With Advanced Pancreatic Cancer: A Prospective Large‐Cohort Study
Source: MedComm (2020). 2026 Jun 23;7(7):e70829. doi: 10.1002/mco2.70829 (PMC13287970; doi:10.1002/mco2.70829)
Supplement: Supplementary file 1 — Figure S1: Study flow chart. PDAC, pancreatic ductal adenocarcinoma. Figure S2: Impact of ctDNA status on PFS and OS. Figure S3: Prognostic impact of ctDNA on survival outcomes in external validation cohort. Figure S4:Impact of clinical characteristics and CA19‐9 on PFS and OS. Figure S5:Clinical utility of ctDNA in CA19‐9‐negative PDAC patients. Figure S6:Impact of B1 ctDNA on PFS and OS by tumor‐naive ctDNA assays. Figure S7. Prognostic significance of ctDNA‐identified somatic mutations. Figure S8:Assessment of normality for ctDNA level and log2 (count+1) using Q‒Q (A) and P‐P (B) plots. Table S1: Baseline characteristics stratified according to ctDNA status. [file MCO2-7-e70829-s001.docx]

**Supplementary Information**

**Dynamics of Circulating Tumor** **DNA (ctDNA) Predicts Treatment Efficacy and Prognosis in Patients with Advanced Pancreatic Cancer: A Prospective Large-cohort Study**

Running title: ctDNA Dynamics in Advanced Pancreatic Cancer

Tingting You^1,2*^, Hui Tang^1,2*^, Mingming Yuan^3^, Dongfeng Song^1^, Chenyu Wang^1^, Jinrong Ying^1^, Rongrong Chen^3^, Chunmei Bai^1^,^#^ Yuejuan Cheng^1#^, Yingyi Wang^1#^

^1^ Department of Medical Oncology, Peking Union Medical College Hospital, Chinese Academy of Medical Sciences, Beijing, 100032, China

^2^Department of Internal Medicine, Peking Union Medical College Hospital, Chinese Academy of Medical Sciences, Beijing, 100032, China

^3^ Geneplus-Beijing, Beijing, 102206, China

* These authors contributed equally to this work.

**Correspondence**

Chunmei Bai, Yuejuan Cheng and Yingyi Wang, Department of Medical Oncology, Peking Union Medical College Hospital, Chinese Academy of Medical Sciences, No.41 Damucang Hutong, Xicheng District, 100032, China.

Email: baichunmei1964@163.com; chengyuejuan@pumch.cn; wangyingyi@pumch.cn.

**
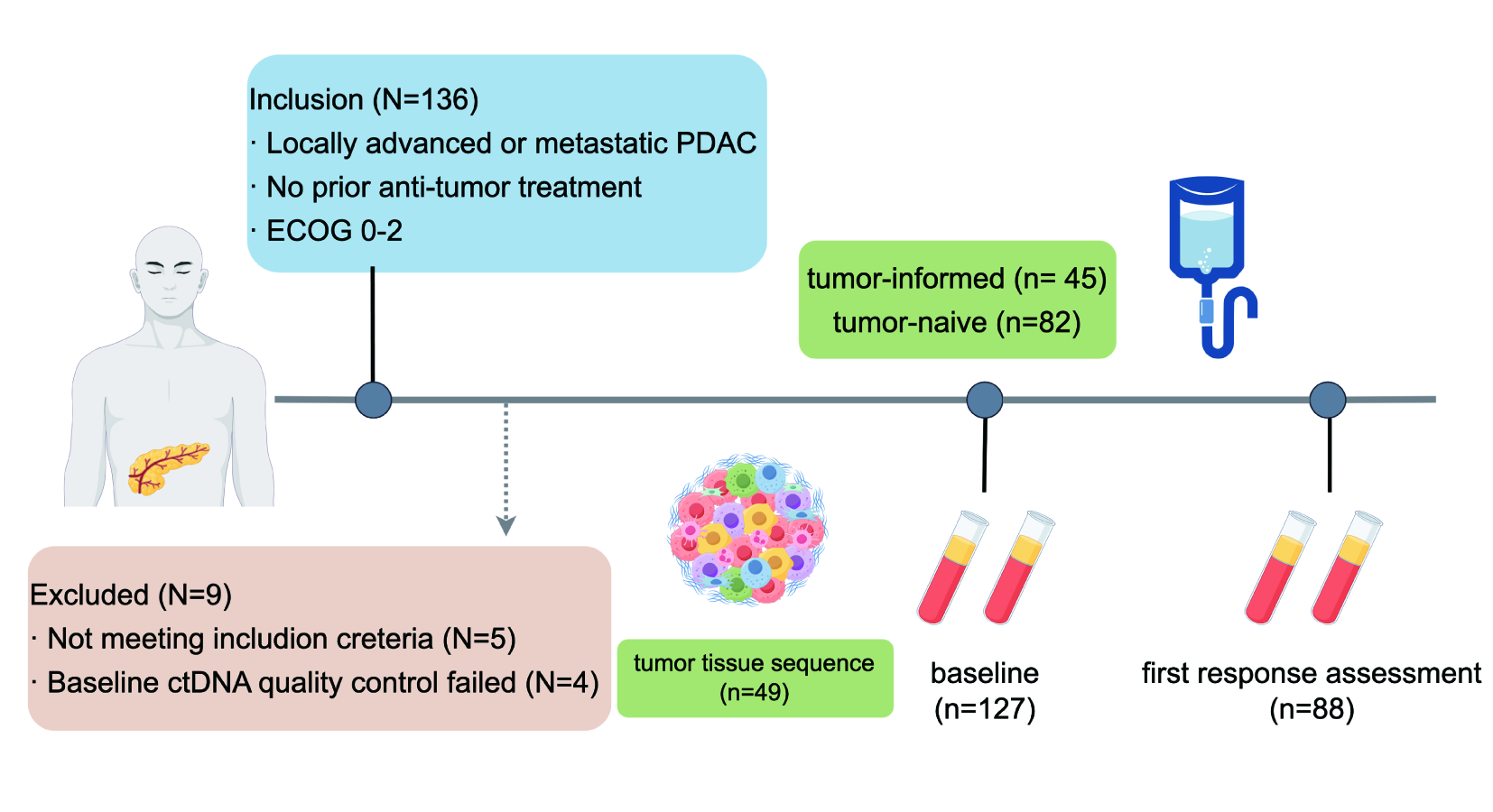
**

**Figure S1. Study flow chart**. Abbreviations: PDAC, pancreatic ductal adenocarcinoma

**
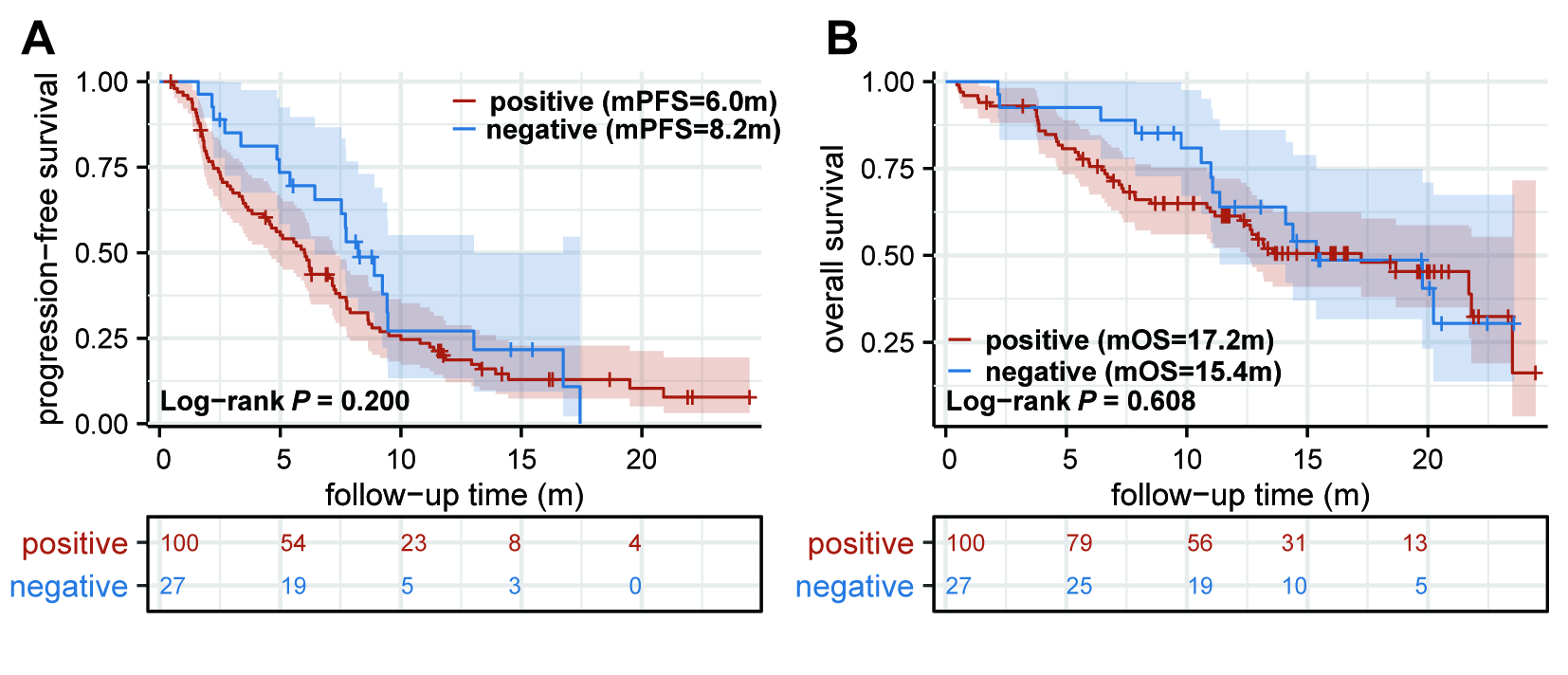
**

**Figure S2**. **Impact of ctDNA status on PFS and OS.**

1. B) Impact of B1 ctDNA status on PFS (A) and OS (B).


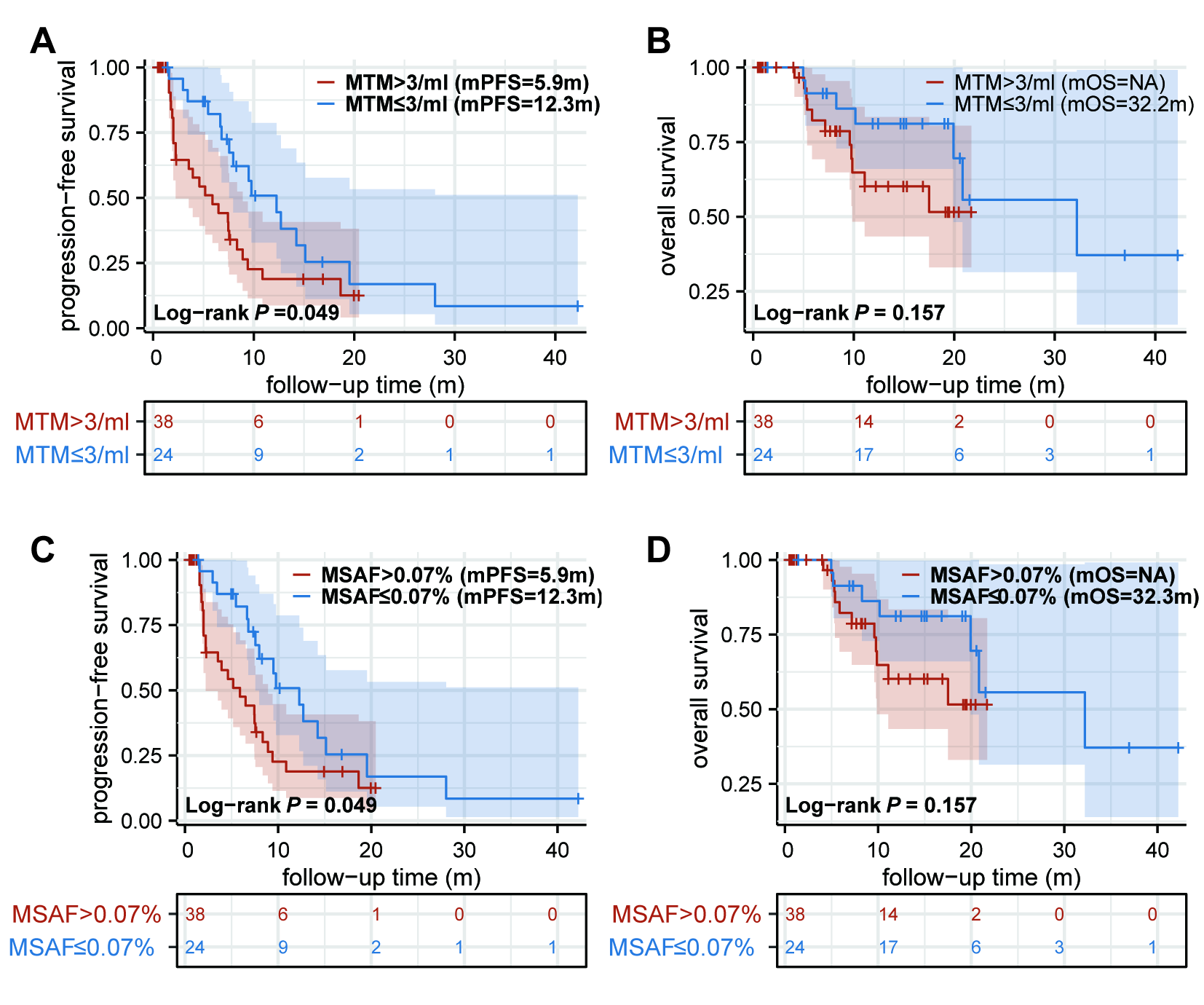


**Figure 3. Prognostic impact of ctDNA on survival outcomes in external validation cohort.**

(A-B) Impact of ctDNA level on PFS (A) and OS (B) stratified by MTM 3/ml in external validation cohort.

(C-D) Impact of ctDNA MSAF on PFS (A) and OS (B) stratified by MSAF 0.07% in external validation cohort.


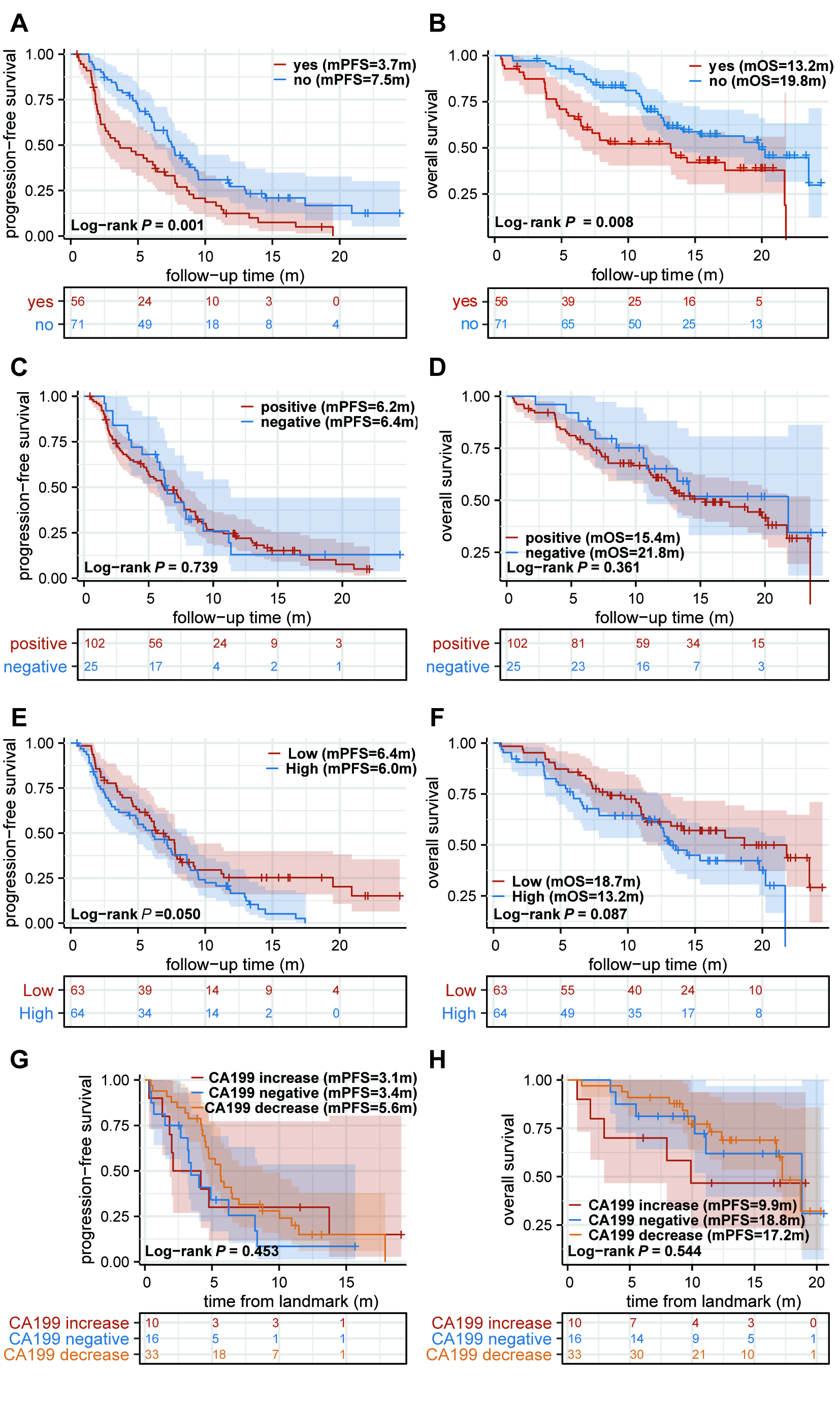


**Figure S4**. **Impact of clinical characteristics and CA199 on PFS and OS.**

(A-B) Impact of liver metastases on PFS (A) and OS (B).

(C-D) Impact of the B1 CA199 status on PFS (C) and OS (D) stratified by the median CA199 level.

(E-F) Impact of the B1 CA199 level on PFS (E) and OS (F) stratified by the median CA199 level.

(G-H) Impact of CA199 changes on PFS (G) and OS (H) by landmark survival analysis.


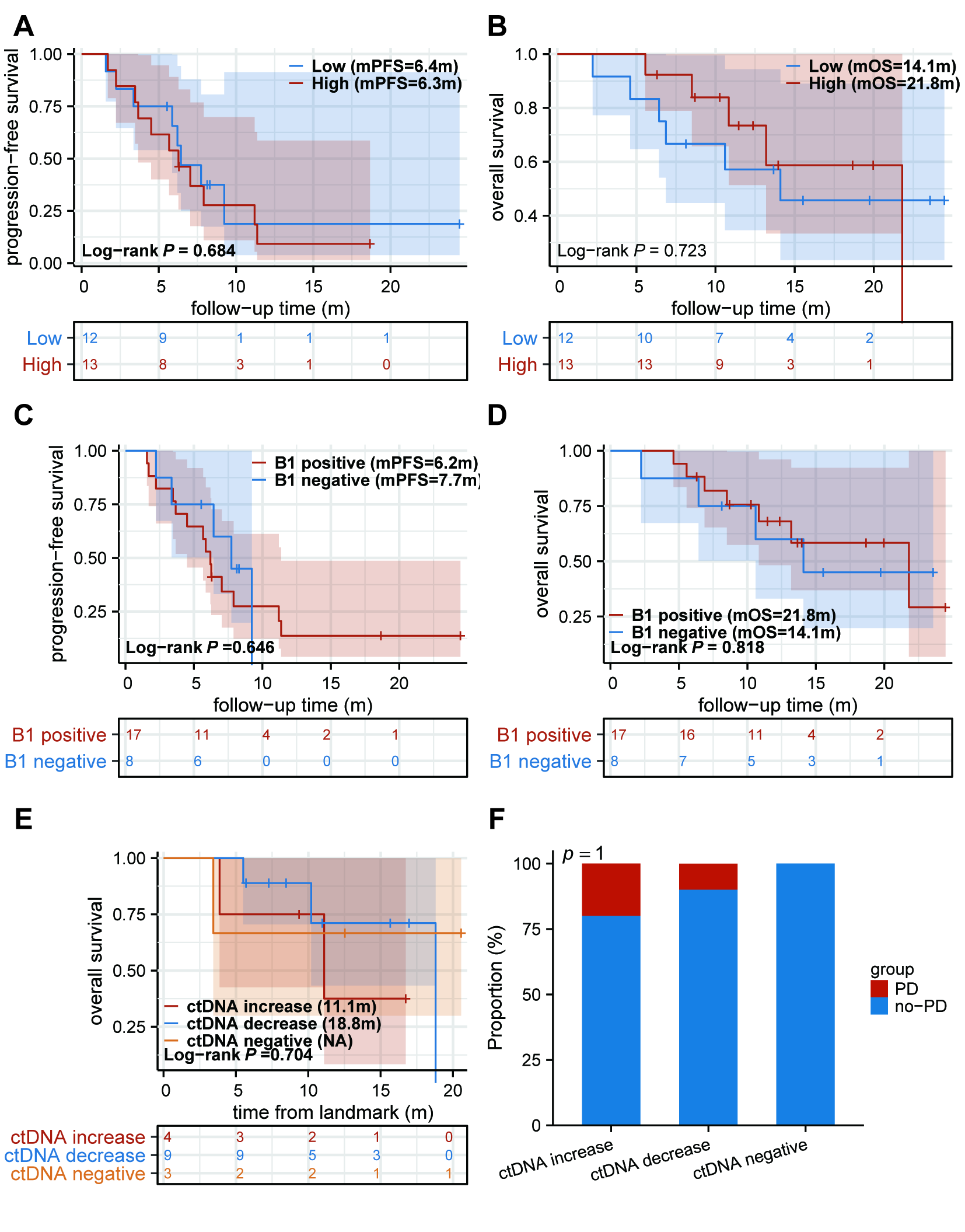


**Figure S5**. **Clinical utility of ctDNA in CA199-negative PDAC patients.**

1. B) Impact of the B1 ctDNA level on PFS (A) and OS (B) stratified by the median ctDNA concentration in the CA199-negative cohort.

(C-D) Impact of B1 ctDNA status on PFS (C) and OS (D) in the CA199-negative cohort.

(E) Impact of ctDNA changes on OS in the CA199-negative cohort by landmark survival analysis.

(F) Correlations between ctDNA changes and the therapeutic response to first-line chemotherapy in the CA199-negative cohort.


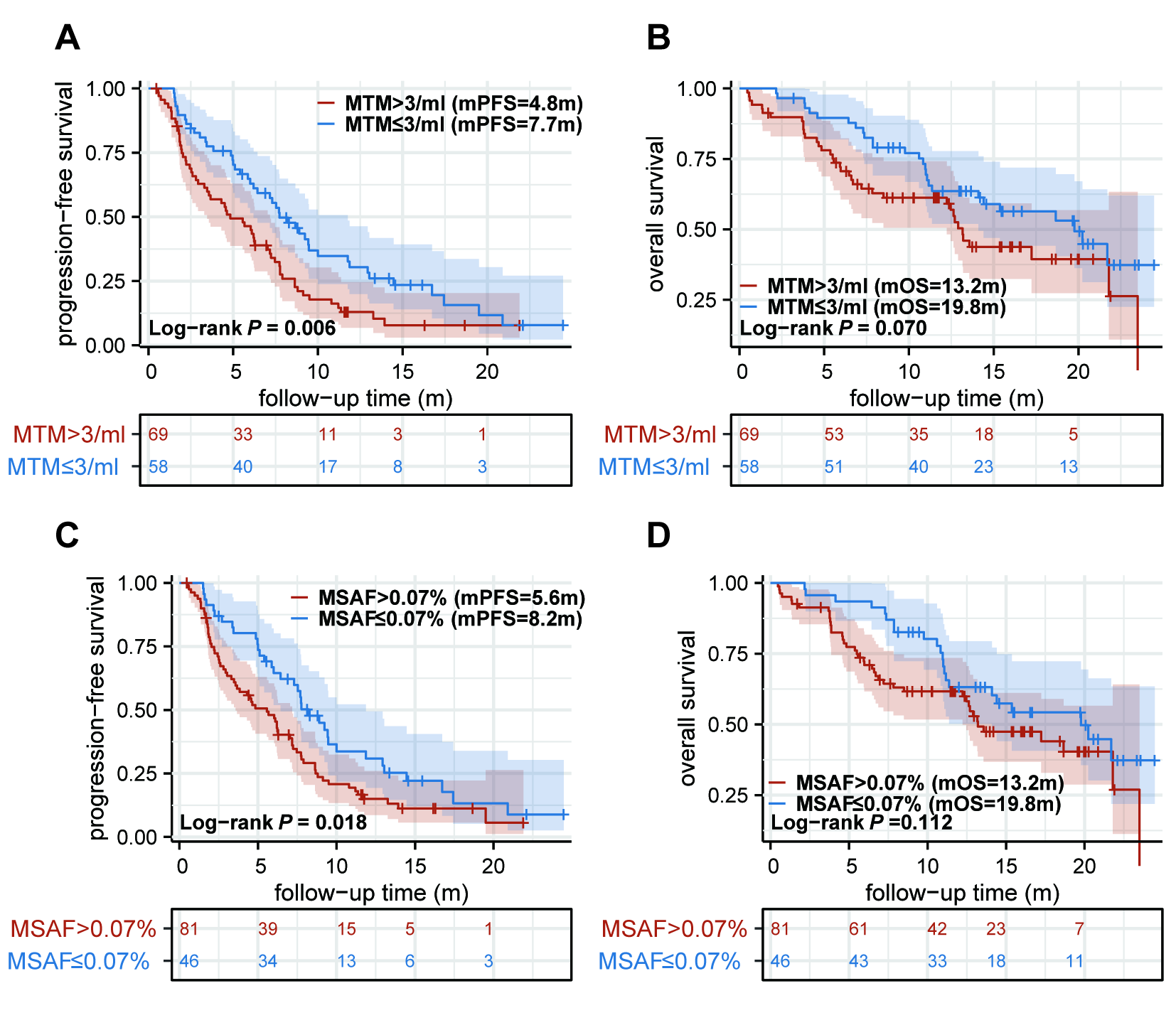


**Figure S6**. **Impact of B1 ctDNA on PFS and OS by tumor-naive ctDNA assays.**

(A-B) Impact of B1 ctDNA level on PFS (A) and OS (B) stratified by MTM 3/ml.

(C-D) Impact of the B1 ctDNA MSAF on PFS (C) and OS (D) stratified by the MSAF 0.07%.


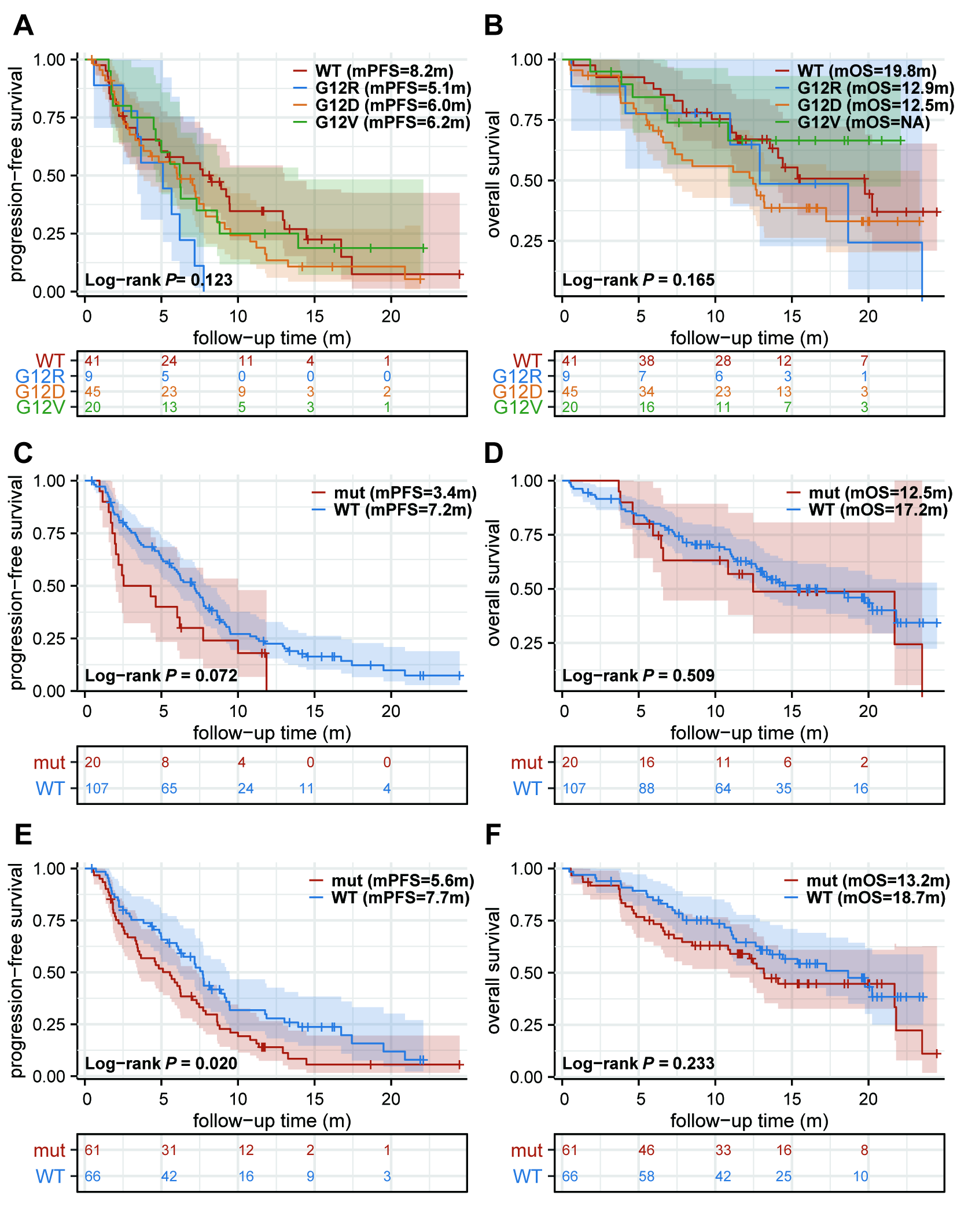


**Figure S7**. Prognostic significance of ctDNA-identified somatic mutations.

(A-B) Impact of KRAS mutation on PFS (A) and OS (B).

(C-D) Impact of CDKN2A mutation on PFS (C) and OS (D).

(E-F) Impact of TP53 mutation on PFS (E) and OS (F). WT,wild type; mut, mutated type


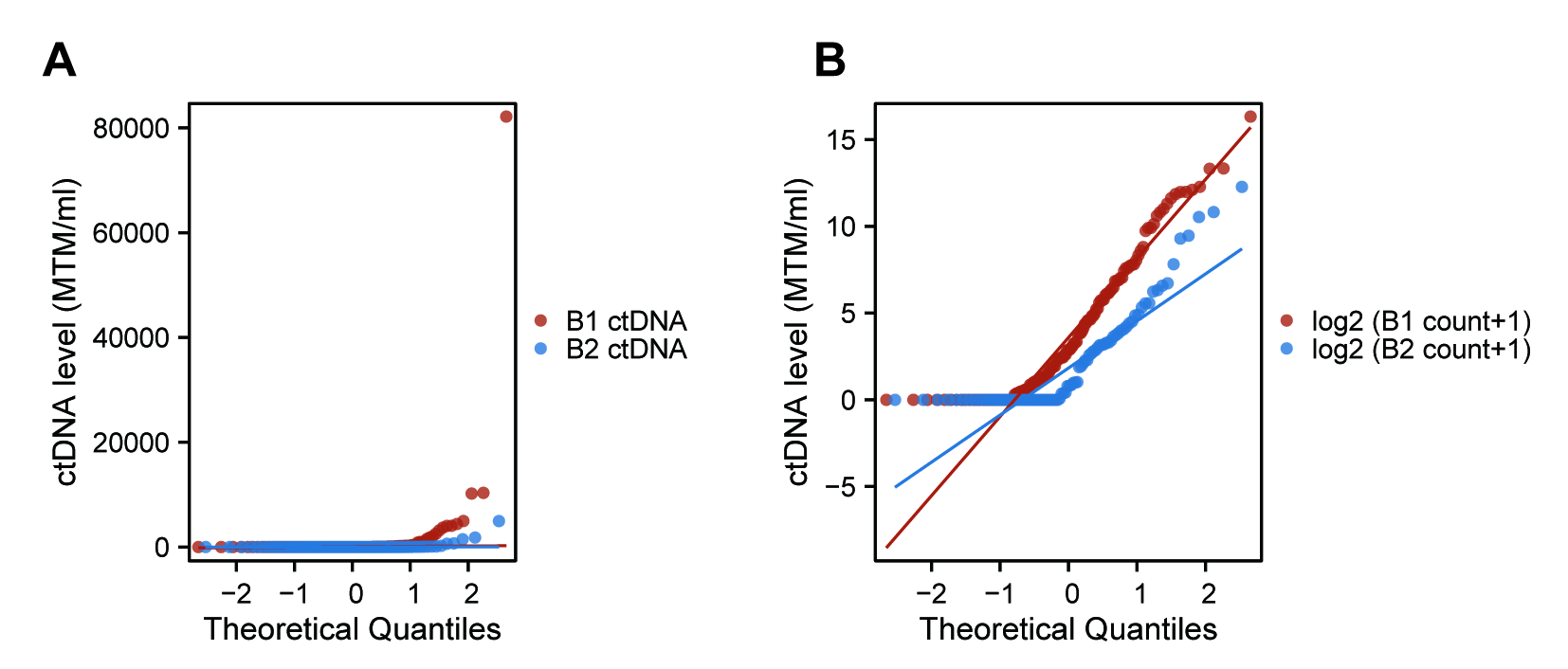


**Figure S8**. **Assessment of normality for ctDNA level and log2 (count+1) using Q‒Q (A) and P-P (B) plots.**

**Table S1. Baseline characteristics stratified according to ctDNA status**

| characteristics | All patients (N=127) | ctDNA positive (N=101） | ctDNA negative （N=26） | P value |
| --- | --- | --- | --- | --- |
| Age | 60.598 ± 9.3836 | 61.29 ± 8.931 | 58.037 ± 10.693 | 0.11 |
| Sex |  |  |  | 0.96 |
| M | 70 (55.1%) | 55 (43.3%) | 15 (11.8%) |  |
| F | 57 (44.9%) | 45 (35.4%) | 12 (9.4%) |  |
| Primary tumor location |  |  |  | **0.00** |
| Head & neck | 57 (45.2%) | 38 (30.2%) | 19 (15.1%) |  |
| Body & tail | 69 (54.8%) | 61 (48.4%) | 8 (6.3%) |  |
| Clinical T stage |  |  |  | 0.64 |
| T1-2 | 41 (32.3%) | 32 (25.2%) | 9 (7.1%) |  |
| T3 | 27 (21.3%) | 23 (18.1%) | 4 (3.1%) |  |
| T4 | 59 (46.5%) | 45 (35.4%) | 14 (11%) |  |
| Clinical N stage |  |  |  | 0.45 |
| N0 | 24 (18.9%) | 16 (12.6%) | 8 (6.3%) |  |
| N1 | 18 (14.2%) | 15 (11.8%) | 3 (2.4%) |  |
| N2 | 10 (7.9%) | 8 (6.3%) | 2 (1.6%) |  |
| Nx | 75 (59.1%) | 61 (48%) | 14 (11%) |  |
| Stage |  |  |  | **0.03** |
| metastatic | 75 (59.1%) | 64 (50.4%) | 11 (8.7%) |  |
| locally advanced | 52 (40.9%) | 36 (28.3%) | 16 (12.6%) |  |
| B1 CA199 (U/ml) | 422.3 (75.15, 1404) | 559.8 (95.1, 1920.8) | 216 (16.15, 494.4) | **0.01** |
| ECOG |  |  |  | 0.26 |
| 0 | 63 (49.6%) | 52 (40.9%) | 11 (8.7%) |  |
| 1 | 60 (47.2%) | 44 (34.6%) | 16 (12.6%) |  |
| 2 | 4 (3.1%) | 4 (3.1%) | 0 (0%) |  |
| Chemotherapy regimens |  |  |  | 0.73 |
| AG | 80 (62.9%) | 64 (50.8%) | 16 (12.7%) |  |
| FFX | 24 (18.9%) | 18 (14.3%) | 6 (4.8%) |  |
| other | 20 | 17 (13.4%) | 5 (3.9%) |  |
| Liver metastases |  |  |  | **0.00** |
| yes | 56 (44.1%) | 51 (40.2%) | 5 (3.9%) |  |
| no | 71 (55.9%) | 49 (38.6%) | 22 (17.3%) |  |
| Lung metastases |  |  |  | 0.85 |
| yes | 13 (10.2%) | 11 (8.7%) | 2 (1.6%) |  |
| no | 114 (89.7%) | 89 (70.1%) | 25 (19.7%) |  |
| Peritoneal Metastasis |  |  |  | 1.00 |
| yes | 11 (86.6%) | 9 (7.1%) | 2 (1.6%) |  |
| no | 116 (91.3%) | 91 (71.7%) | 25 (19.7%) |  |

Characteristics were presented as median (range), mean ± SD or N (%) according to their data type and distribution.

Tested by Chi-square test, T test, Wilcoxon test or Yates' correction.

Abbreviations: M-PDAC, metastases pancreatic ductal adenocarcinoma; LA-PDAC, locally advanced pancreatic ductal adenocarcinoma; ECOG, Eastern Cooperative Oncology Group; AG: nab-paclitaxel + gemcitabine; FFX: leucovorin + 5-FU + oxaliplatin + irinotecan.
